# Biocompatibility of three bioabsorbable membranes assessed in FGH fibroblasts and human osteoblast like cells culture

**DOI:** 10.1186/1746-160X-10-29

**Published:** 2014-08-06

**Authors:** Michelle Pereira Costa Mundim Soares, Paulo Vinícius Soares, Analice Giovani Pereira, Camilla Christian Gomes Moura, Priscila Barbosa Ferreira Soares, Lucas Zago Naves, Denildo de Magalhães

**Affiliations:** 1 Master degree of Dentistry School of Federal University of Uberlandia, Uberlandia, Minas Gerais, Brazil; 2 Associate Professor of Operative Dentistry and Dental Materials Department at the Dentistry School of Federal University of Uberlandia, Uberlandia, Minas Gerais, Brazil; 3 Doctoral student of Dentistry School of Federal University of Uberlandia, Uberlandia, Minas Gerais, Brazil; 4 Adjunct Professor of Federal University of Triangulo Mineiro, Uberaba, Minas Gerais, Brazil; 5 Post Doctoral student of Dentistry School of Estadual University of Campinas, Piracicaba, São Paulo, Brazil; 6 Associate Professor of Histology of Periodontics and Implant Dentistry Department at the Dentistry School of Federal University of Uberlandia, Av. Para 1720, Campus Umuarama, Uberlandia, Minas Gerais, Zip Code 38400-000, Brazil

**Keywords:** Membranes, Biosorbable, Regeneration, Fibroblast, Osteoblast

## Abstract

**Objectives:**

Specific physical and chemical features of the membranes may influence the healing of periodontal tissues after guided tissue regeneration (GTR). The aim of the present investigation was to analyze the biological effects of three bioabsorbable membranes. The hypothesis is that all tested membranes present similar biological effects.

**Methods:**

Human osteoblast like-cells (SaOs-2) and gingival fibroblasts FGH (BCRJ -RJ) were cultured in DMEM medium. The viability of the cells cultured on the membranes was assesses using 3-(4,5-Dimethylthiazol-2-yl)-2,5-diphenyltetrazolium bromide (MTT). Quantitative determination of activated human Transforming Growth Factor beta 1 (TGF-β1) on the supernatants of the cell culture was observed. Samples were examined using scanning electron microscope (SEM).

**Results:**

SaOs2, in 24 hours, PLA group showed higher values when compared to other groups (P < 0.05). All groups presented statistical significance values when compared two times. In 4 h and 24 h, for the fibroblasts group, significantly difference was found to PLA membrane, when compared with the other groups (p < 0.05). For TGFβ1 analyzes, comparing 4 and 24 h, for the osteoblast supernatant, COL1 and PLA groups showed statistically significant difference (p <0,008). On the analysis of culture supernatants of fibroblasts, in 24 hours, only PLA group presented significant difference (p = 0,008).

**Conclusions:**

The biomaterials analyzed did not show cytotoxicity, since no membrane presented lower results than the control group. PLA membrane presented the best performance due to its higher cell viability and absorbance levels of proliferation. Both collagen membranes showed similar results either when compared to each other or to the control group.

## Background

Periodontal disease is very common in general population and is the major cause for loss of the tooth-supporting apparatus [1]. The goals of periodontal therapy have been described in many ways over the years. The key concept is to improve periodontal health and thereby to satisfy a patient’s aesthetic and functional needs or demands [2,3]. Regeneration of the damaged structures using regenerative procedures present variable success rates, depending on multiple factors such as defect size and type, patients’ age, genetics [1].

Regeneration is defined as the reproduction or reconstitution of a lost or injured part [4]. Periodontal regeneration requires restitution of the periodontal attachment apparatus including new bone formation, new cementum deposition upon the denuded root surface, and reinsertion of functionally oriented new collagen fibers of the periodontal ligament, into the new bone and new cementum [1,5]. Cementoblasts, fibroblasts, and osteoblasts and their precursors are the principal cells found in periodontal ligament [2,6,7]. Physical barriers can be applied during regeneration procedures to exclude unwanted cells from the wound space to promote periodontal regeneration. The desirable characteristics of barrier membranes include biocompatibility, cell occlusion properties, integration by the host tissues, ability to induce cellular proliferation and differentiation, clinical manageability and space making ability [3].

The use of barrier membranes has become a standard step on guided tissue regeneration (GTR) introduced in 1988, by Dahlin, as a therapeutic modality aiming, excluding epithelial and connective tissues, to enable bone progenitor cell proliferation and differentiation into the isolated area [8,9]. The aim of GTR is fully reestablish functional periodontium, including new cementum and periodontal ligament, as well as new bone regeneration. Since fibroblasts from the wound margins are able to attach to the membrane the proliferation of the epithelial cells is stopped in the presence of collagen [10,11].

Various biomaterials from natural and synthetic origin have been extensively investigated about biocompatibility, biodegradability, cell interaction, and mechanical properties. The prepared scaffolds with various shapes and structures have to suitable carry the cell, provide initial support similar to extracellular matrix and facilitate the nutrient and metabolite diffusion during *in vitro* culture. For *in vivo* cultures the membrane is expected to allow angiogenesis after transplantation, ensuring continuous viability and functionality of the regenerative cells [1,11]. Several materials have been tested for their effectiveness as barriers such as millipore filters, expanded polytetrafluoroethylene (ePTFE) membranes, collagen membranes, polygalactin, calcium sulfate and polylactid acid membranes [4,5,12].

A disadvantage of non-absorbable membranes is the need of a second-step surgery to remove the membrane. This procedure may injure the newly formed granulation tissue. Furthermore, early spontaneous exposure to the oral environment and subsequent bacterial colonization has been reported to be common problems of non-absorbable membranes [5,13,14]. Barrier materials derived from porcine or bovine collagen type I and III demonstrated their usefulness in GTR procedures. However, several complications such as early membrane degradation, epithelial down growth and premature loss of the material were reported following the use of collagen materials [5]. Besides the surgical aspects, specific physical and chemical features of the membranes may influence the healing of periodontal tissues after GTR therapy [15].

The purpose of the present investigation was to analyze the biological effects of commercially available bioabsorbable collagen and polylactic acid membranes in cultures of gingival fibroblasts, and human osteoblast-like cells. In particular, to analyze the proliferations rate/cell viability, TGFβ1 level and the adhesion/morphology of the cells in contact to the membranes by scanning electron microscopy (SEM).

## Methods

The present study was performed as mastering dissertation at School of Dentistry of Federal University of Uberlandia under the approval of Post Graduate degree Program.

### Membranes examined

Three commercially available bioabsorbable membranes with different composition and structures were examined: Gore Tex (polylactic acid - Resolut W L Gore and Associates Inc Flagstaff A Z), Gen Derm (type I bovine collagen – Genius biomaterials – Baumer SA Brazil), Surgidry Dental F (type I bovine collagen – TechoDry Liofilizados Médicos Ltda Brazil) (Table 1).

**Table 1:** Groups and membranes used in the study

**Group description**	**Composition**	**Bioabsorbable membrane**	**Manufacturer**
**PLA**	POLYLACTIC ACID	GORE TEX	W L Gore and Associates Inc Flagstaff A Z
**CL1**	TYPE I BOVINE COLLAGEN	GEN DERM	Baumer SA Brazil
**CL2**	TYPE I BOVINE COLLAGEN	SURGIDRY DENTAL F	TechoDry Liofilizados Médicos Ltda Brazil

### Cell cultures

A SaOs-2 cell line and gingival fibroblasts FGH, immortalized cell line obtained from the Banco de Células do Rio de Janeiro (BCRJ-RJ)were cultured in a humidified atmosphere (95% air, 5% CO _2_) at 37°C, maintained in DMEM high glicose medium (DMEM, Cultilab, SP, Brazil) containing gentamicine sulfate and anfotericina B, supplemented with 10% fetal bovine serum. Tissue culture medium was changed every 2 days until confluence was reached. Upon reaching confluence, the cells were detached using trypsin-EDTA solution. Cells between the passages 2-3 were counted for viability using Trypan Blue.

The membranes (diameter 3,65 mm) were placed in 96-well plates and immersed in serum-free medium for 15 min. Then the medium was discarded and 1×10^5^ SaOs-2 were seeded in the well plates. Cells plated on the well without barriers served as positive control. The same was carried for the fibroblasts FGH cells.

### Viability test

The viability of the cells cultured on the membranes was assessed using 3-(4,5-Dimethylthiazol-2-yl)-2,5-diphenyltetrazolium bromide, a yellow tetrazole (MTT). The adhesion test was carried out in according to Mosmann [16]. The membranes were removed from the wells after 4 and 24 h, 100 μl of 1% MTT was added and then incubated at 37°C for 3 h. After incubation, the MTT containing medium was removed from the plate and 100 μl of solubilizing solution, consisting of 20% of Sodium lauryl sulfate in 50% dimethylformamide, was added to each well. This addition was performed to dissolve the formazan crystals formed from the tetrazolium salts. The optical density (OD) of the colored complex formed was read by spectrophotometer with 570 nm wavelength. The amount of viable cells adhered to the membranes (directly proportional to OD) was calculated by Digiread software based on the resultsobtained from the spectrophotometer. The experiments were performed five times for each sample. The Analysis of Variance Two-way test and Tukey’s test (p < 0.05) were applied.

### TGFβ1 Level

For quantitative determination of activated human Transforming Growth Factor beta 1 (TGF-β1) concentrations in cell culture supernatants ELISA test (Human/Mouse TGFβ 1 ELISA Ready-SET-Go! -eBioscience, San Diego,USA) was performed.

The cytokine TGFβ1 was assessed to indicate the presence of growth factor. ELISA plates were used for high-affinity. The plates were sensitized with anti-TGFβ1, kept overnight in refrigerator and then washed with PBS-Tween 5 times. The sites were blocked for 1 hour rinsed with PBS-T 3 times. The second detection antibody was added for 1 h washed with PBS-T 5 times, added AVIDIN HRP for 30 minutes, washed with PBS-T 5 times. Then Tetramethylbenzidine solution (TMB) was added. The optical density was determined using a microplate reader set to 450 nm. The results were compared with a wavelength control curve. The Kruskal Wallis One Way Analysis and Mann Whitney test (p < 0.05) were employed.

### Scanning electron microscopy examination

Three membranes of each group was fixed and processed for electron microscopy 24 h after culture. Fixation was carried out with 2,5% glutaraldehyde at pH7,2 for 4 h. The fixed samples were washed twice with phosphate buffered saline (PBS) for 5 minutes each followed by immersion in 1% osmium tetra oxide, for 1 h. Then the samples were washed twice with PBS followed by serial dehydration in series of graded ethanol solutions ranging from 50 to 100%; dried over 15 minutes with 50% vol/vol mixture of hexamethyldisilazane (HMDS) and ethanol, and finally with 100% HMDS twice for 15 minutes. Finally the samples were air dried by leaving them partly covered for 4 h, mounted onto 12.5-mmdiameter carbon tabs and aluminum stubs and gold sputtered with 20 nm. Samples were examined using a scanning electron microscope (SEM).

## Results

### Viability test

**Figure 1:**
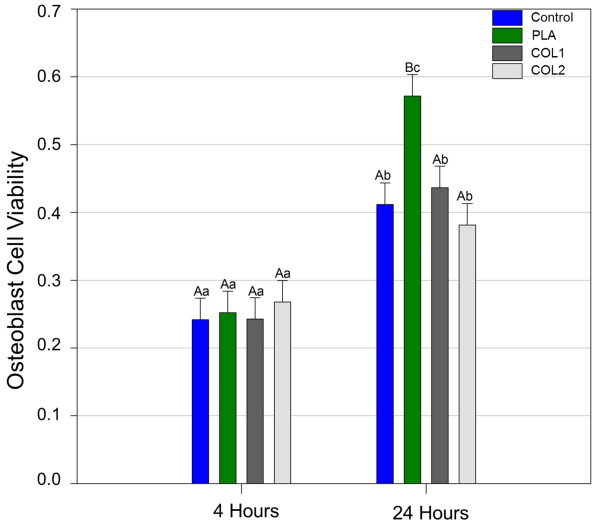
Results of MTT test for osteoblast cells.

**Figure 2:**
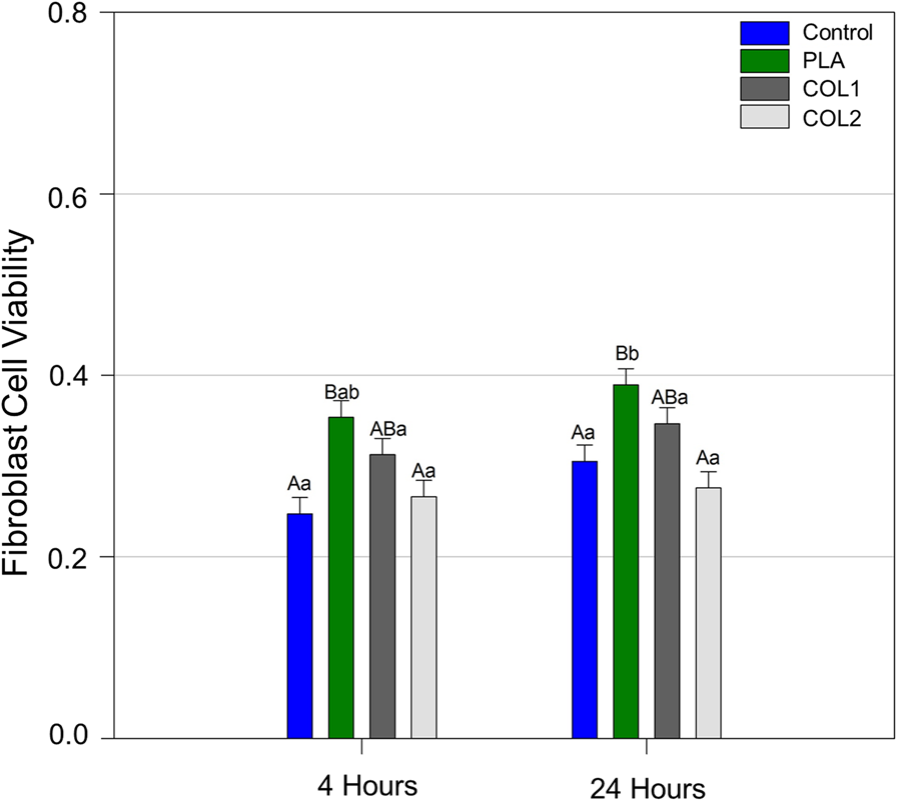
Results of MTT test for fibroblast cells.

Statistically significant differences were found between the periods 4 and 24 h evaluated for all osteoblasts groups. There was no statistical difference between the osteoblasts groups when compared in 4 h. For 24 h the PLA group showed significantly higher values when compared to the other groups (P <0.05) (Figure 1). From 4 to 24 h of culture, the MTT test did not show increasing metabolic activity of the fibroblasts in all groups of membranes. In 4 h, for the fibroblasts group, significantly difference was found for PLA membrane compared with control (p < 0,001), and PLA with COL2 (p = 0,006). In 24 h analysis, PLA presented significant difference when compared to COL2 (p < 0,001) and control (p = 0,010) (Figure 2).

### TGFβ1 level

Comparing two times, 4 and 24 h, with the osteoblast supernatant, COL1 and PLA groups showed statistically significant difference (p <0.008). For 4 h COL1 and PLA groups showed significantly higher values when compared with control and COL2 (p <0.05). For 24 h the group COL2 showed significantly lower values when compared with other groups (Table 2). On the analysis of culture supernatants of fibroblasts, 4 and 24 h, only PLA group presented significant difference (p = 0.008). For 4 h all the groups showed similar results and for 24 h PLA group showed higher significant values when compared to other groups (Table 3).

**Table 2:** Results of ELISA test for osteoblast cells

**Time**	**Control**	**PLA**	**COL1**	**COL2**
4 hours	147,8 (0,3) Ba	4347,5 (723,5) Aa	5715,0 (1391,7) Aa	147,7 (0,8) Ba
24 hours	147,6 (0,50) Aa	147,9 (0,12) Ab	147,7 (0,10) Ab	147,7 (0,09) Ba

Uppercase letters indicate differences in the lines, lowercase letters indicate differences in columns.

**Table 3:** Results of ELISA test for fibroblast cells

**Time**	**Control**	**PLA**	**COL1**	**COL2**
4 hours	148,0 (1,10) Aa	148,0 (0,36) Aa	147,8 (0,12) Aa	147,7 (0,34) Aa
24 hours	148,0 (0.63) Aa	2972,9 (777,61) Ba	148,9 (0,29) Aa	148,0 (0.74) Aa

Uppercase letters indicate differences in the lines, lowercase letters indicate differences in columns.

### Scanning electron microscopy

**Figure 3:**
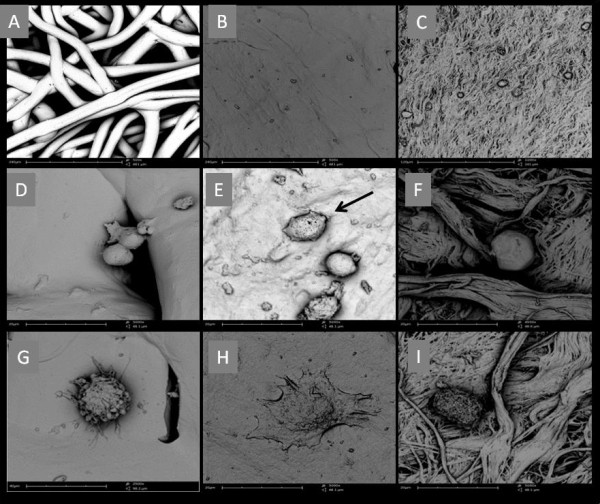
**SEM analysis. (A)** PLA membrane showing separation between the fibers. **(B)** COL1 surface showing more regular structure with some pores. **(C)** COL2 showing woof of small fibers and more layers. **(D)** Attachment time 24h osteoblast cell beginning to emit small cytoplasmic expansion in PLA. **(E)** Attachment time 24h COL1 and some rounded SaOs-2 cell with initial lamellopodia represented with arrow in the image. **(F)** Attachment time 24h COL2 revealing rounded osteoblast. **(G)** Attachment time 24h PLA showing fibroblast adhesion with cytoplasm expansion **(H)** Attachment time 24h COL1 fibroblasts were also observed flatted, beginning to look like a star format. **(I)** Attachment time 24h COL2 round shaped fibroblasts were observed.

SEM examination showed attachment and physiologic morphology grown of the cells on the membranes. After 24 h culture SEM showed that both cells had attached to all groups of membranes. However, some cells had flattened onto the collagen membrane while those on PLA remained rounded. SEM examination of cells cultures revealed healthy cells populations (Figure 3).

## Discussion

The use of GTR has proved to be a suitable technique when simulating regeneration, new connective tissue attachment and bone formation [17]. When a membrane is placed between the denuded root surface and the repositioned mucogingival flap, it provides a secluded space into which fibroblasts and osteogenic cells from the healthy apical portion of the periodontium may migrate [15].

The purpose of this study was to determine the biocompatibility of several barrier materials in human cell cultures, which are comparable to the periodontal regenerative cells. The cells selected for this study in vitro, were gingival fibroblasts and osteoblasts, since both are present in periodontal tissue regeneration.

The viability cell test showed that the absorbance level for osteoblasts increased for all groups between 4 and 24 h, indicating that the membranes did not exhibit cytotoxicity to cells. The PLA group showed better results when compared to other groups at 24 h. The cells probably reached the log phase showing higher viability and metabolic activity.

In the analysis of fibroblasts proliferation of using the MTT method, the membrane PLA, also presented better results than the other groups. However, in 24 h analysis it was observed that COL1 and COL2 were similar to PLA. Fibroblasts and osteoblasts have different growth kinetics. Since fibroblasts have reached the log phase at 24 h assessment the absence of statistical difference between the groups between 4 and 24 h can be confirmed by the same behavior observed in the control group. Probably the characteristics of the PLA membrane favor cell proliferation.

The TGFβ1 test confirmed the ability of proliferation of both cells fibroblasts and osteoblasts. When in contact to PLA membrane a statistical significant difference was observed for two types of cells. For COL1 group the osteoblasts dosage of the supernatant were significant. Probably, the contact with the membranes further favors cell proliferation, since it was observed in greater proportion than in control group. The TGFβ1 is a proliferation factor that acts in the repair process. Besides assessing cell viability, the aim of the study also includes analysis of the cells ability to regenerate when in contact with the membranes. Another important factor is that TGFβ1 is present in the two types of cells evaluated.

SEM analysis showed that the cells had a rounded and flattened appearance on collagen membrane, associated with cellular health. The cells migrated horizontally on the collagen membrane. However, this pattern was not seen in PLA membrane, because of the high distance among the fibers that permitted cells to be in direct contact with only an individual fiber. SEM examination of the fibroblasts cells on the collagen revealed cytoplasmatic membranes and lamellipodia extension presence. Also the surface topography of the membranes plays an important role in cell’s adhesion, rough or porous textured surfaces have been considered to promote attachment cell. Morphology can be regarded as an indicator of the affinity of the cells to a substratum [18].

The adhesion of mesenchymal cells to barrier membranes is dependent of the type and nature of the barrier and is thought to influence regenerative results positively [10]. These barrier membranes must, ideally, present the ability to allow cell’s adhesion and induce cellular proliferation and differentiation [10].

The results of this study indicate that biomaterials do not affect the cellular proliferation, once all results were similar or better than control group. In the present study the results showed that collagen membrane presented lower values than PLA membrane which are similar to Chandrahasa, et al. 2011 [19], Parrish et al. 2009 [4] and [14]. Polylactic acid and collagen membranes’ biocompatibility was evaluated in vitro using osteoblast-like cells. The results showed proliferation capacity in both types of materials, but better results were found polylactic acid membrane PLA group [14]. In a systematic review, it was observed that the use of polylactic acid membrane even shows better results than open flap debridement with grafts [4].

The results of all experiments indicated that PLA membrane exhibited excellent biocompatibility in contradiction with other in vitro studies using polylactic acid membranes [3,5,20]. Collagen membranes have shown regenerative favorable results, due to their excellent biocompatibility and cell affinity. Nevertheless, collagen based membranes show relatively poor mechanical and dimensional stability, due to its rapid degradation [10]. Among the papers of a systematic literature review performed to evaluate collagen membranes on the potential of different cells to attach to, proliferate on, and migrate over barrier membranes, an in vitro study showed that different cell types have different comportments on identical membranes [11].Another factor that must be considered is that, despite having the same composition, biomaterials and treatments may have different morphologic structures (Figure 3). So, caution is essential while interpreting results obtained by using in vitro experimental model, since cell type, material characteristics and patient response may play an important role.

Further studies using controlled experimental models in vivo are needed in order to verify the present results. Guided tissue regeneration has different degrees of success, depending on the type of barrier selected, presence or absence of underlying graft material, type of graft, feasibility of the technique applied, and clinician’s preference among other factors [13].

## Conclusion

Within the limits of this study, it was concluded that biomaterials did not show cytotoxicity, since no membrane showed lower results than the control group. PLA membrane presented it’s the highest biocompatibility and absorbance levels of proliferation and COL1 membrane showed similar results for the test with fibroblasts.

## Competing interests

The authors declare that they have no competing interests.

## Authors’ contributions

DM and PBFS made substantial contributions to conception of the present study. MPCMS and CCGM performed analysis and interpretation of data besides drafting the manuscript. LZN had led acquisition of data. PVS and AGP involved in revising the manuscript critically and giving final approval of the version to be published. All authors read and approved the final manuscript.
